# Culturing Periprosthetic Tissues in BacT/Alert® Virtuo Blood Culture Bottles for a Short Duration of Post-operative Empirical Antibiotic Therapy

**DOI:** 10.7150/jbji.44621

**Published:** 2020-05-16

**Authors:** Claire Duployez, Frédéric Wallet, Henri Migaud, Eric Senneville, Caroline Loiez

**Affiliations:** 1 Institute of Microbiology, Lille University Hospital, 59037 Lille, France; 2 Orthopaedic Department, Lille University Hospital, 59037 Lille, France; 3 Infectious Diseases Department, Gustave Dron Hospital, 59200 Tourcoing, France; 4 University Hospital of Lille, 59037 Lille, France

**Keywords:** prosthetic joint infection, post-operative empirical antibiotic therapy, optimal duration of culture

## Abstract

**Introduction:** A post-operative empirical antibiotic therapy (PEAT) is required in periprosthetic joint infections. It commonly uses broad-spectrum antibiotics to cover most Gram-positive cocci and Gram-negative bacilli. It is currently continued until first microbiological results are available, no less than five days later.

**Methods:** We performed a retrospective study in order to evaluate duration of incubation required for surgical samples using the BacT/Alert® Virtuo blood culture bottles system.

**Results:** Among 216 surgical interventions and 199 clinical strains (53.8% staphylococci, 22,1% streptococci and enterococci, 14,6% Gram-negative bacilli, 5,5% anaerobes), 90.5% of the strains were detected between day 0 and day 2; 15 infective strains are cultured from day 3 including 8 *Cutibacterium* sp., 4 staphylococci, 2 streptococci and 1 *Enterococcus*.

**Conclusions:** We suggest that the duration of PEAT in patients operated for a periprosthetic joint infection may be shortened to three days as Gram-negative rods are unlikely to grow after three days of culture by using BacT/Alert® Virtuo blood culture bottles. This is likely to shorten the overall length of hospital stay, to diminish the occurrence of adverse side effects, and the emergence of antimicrobial resistance. However, coverage of Gram-positive cocci should be maintained for 14 days until the definite culture results are available.

## Introduction

Periprosthetic joint infections (PJIs) require both surgical intervention and prolonged course of intravenous and oral antibiotic therapy conducted in light of the most recent guidelines for the management of these potentially life-threatening infections 1,2.

The susceptibility profile of bacteria isolated from the intraoperative samples requires at least 3 to 5 days to become available. Until the results of intraoperative sample cultures are available, an initial broad-spectrum post-operative empirical antibiotic therapy (PEAT) is needed in order to prevent the colonization of newly implants or the prosthesis that has been cleaned but not removed during the so-called debridement antibiotic and implant retention (DAIR) intervention. PEAT needs to cover most Gram-positive cocci (including methicillin-resistant staphylococci) and most Gram-negative bacilli as it is usually difficult to reliably identify the pathogen(s) before the revision even by the means of a joint aspiration. In addition PEAT may favor the emergence of antibacterial resistance but also increases the antibiotic-related adverse effects and the overall cost of the treatment. Moreover, delayed adequate antibiotic treatment of PJI is associated with worse outcomes 2,3.

For these reasons, rapid detection of infection is of paramount importance. Two-week duration of culture is generally proposed to allow isolation of slow-growing organisms 4 but the optimal duration is actually unknown 2. In this context, use of semi-automated methods such as automated blood culture system may be an attractive alternative to enrichment broths: they contain antibiotic adsorbent, and do not require daily inspection, source of contamination. We set up in our laboratory the culture of these samples in aerobic and anaerobic bottles incubated in the BacT/Alert® Virtuo blood culture bottles system. The main objective of the present study was to evaluate duration of incubation required using the BacT/Alert® Virtuo blood culture bottle system to obtain microbiological results for diagnosis of PJIs, regardless of their sensitivity and specificity. The second objective was to determinate what is the minimum duration of PEAT until reliable microbiological results are available.

## Methods

### Definitions

PJIs were diagnosed in accordance with the Infectious Diseases Society of America definition 2.

### Study design and population

This retrospective study was performed at the French National Reference Centre for Complex Osteoarticular Infections in the North West region of France (CRIOAC Lille-Tourcoing France). Medical charts of all adult patients with documented PJI who received PEAT from January 2018 to December 2018 were reviewed. All patients included in this study had surgical management including DAIR and one or two-stage replacement.

### Surgical management and curative antibiotic therapy

All surgical procedures were performed without antibiotic prophylaxis in patients who had not received antibiotics within two weeks before the intervention 2,5. In accordance with our epidemiological data 6, PEAT consisting with ceftobiprole, or a combination of cefepime and daptomycin as first-line choices, were started intravenously as soon as perioperative samples were taken. According to our current protocol, PEAT was continued until the first results of intraoperative samples cultures were available, i.e the fifth day after surgery, and was then modified in accordance with the culture results (culture-based antibiotic therapy).

### Microbiology

During surgical procedures, at least 3 samples were taken from different areas suspected of being infected using a separate sterile instrument for each sample and were sent to the laboratory within 2 hours. The samples were processed in a class 2 biosafety cabinet. Firstly, solid samples were vortexed without sonication in sterile water for one minute (Ultra Turrax®, IKA, Staufen, Germany). Each sample was then plated for five days at 35°C onto Columbia agar with blood 5% and chocolate agar with polyvitex, and for 14 days in aerobic and anaerobic bottles (1 mL in each bottle) in the BacT/Alert® Virtuo blood culture bottle system (BioMérieux, Marcy l'Etoile, France). Strains were identified using MALDI-TOF spectrometry mass (Bruker Daltonics, Wissembourg, France) with a minimal score requirement of 2. The antibiotic susceptibility profile of all pathogens identified from intraoperative samples was assessed either by the Vitek 2 cards (BioMérieux, Marcy l'Etoile, France) or by agar diffusion technique using the procedure and interpretation criteria proposed by the Comité de l'Antibiogramme de la Société Française de Microbiologie (CA-SFM EUCAST 2018) (http://www.sfm-microbiologie.org).

### Ethical considerations

All patients' collected data were anonymized and recorded on a standardized form preventing any personal identification according to procedures defined by the French information protection commission (Commission Nationale de l'Informatique et des Libertés-CNIL).

## Results

### Patients

During the study period, we identified 106 patients managed for PJI (57 hip prostheses, 39 knee prostheses, 9 shoulder prostheses, 4 elbow prostheses and 4 other prostheses). A total of 216 surgical interventions were recorded from these 106 patients. The demographic characteristics of the included patients are reported in Table 1.

### Microbiology

Over the 216 reported surgical procedures, microorganisms grew in blood culture bottles for 149 cases (69.0%). Among these positive surgical procedures, 58 remained sterile on Columbia agar with blood 5% and chocolate agar with polyvitex and 91 were also positive on solid agar cultures and bottles (Table 2).

Among the 149 positive surgical procedures, using these medium, 141 were detected positive within the first five days, accounting for 95.5% of the strains cultured (190/199, including redundant strains sampled from the same patients in different surgeries).

Overall, a total of 199 clinical strains were identified from intraoperative samples (Table 3). *Staphylococcus* spp. accounted for 53.8% of all strains, especially coagulase negative staphylococci (CoNS) (31.7% of all strains). *Streptococcus* spp. and *Enterococcus* spp. accounted for 22.1% of all strains and Enterobacteriales for 12.6%. Among non-fermenting Gram-negative bacilli, 3 strains of *Pseudomonas aeruginosa* and 1 strain of *Acinetobacter baumannii* were identified. At last, for 2 patients, yeasts were identified.

**Figure 1:**
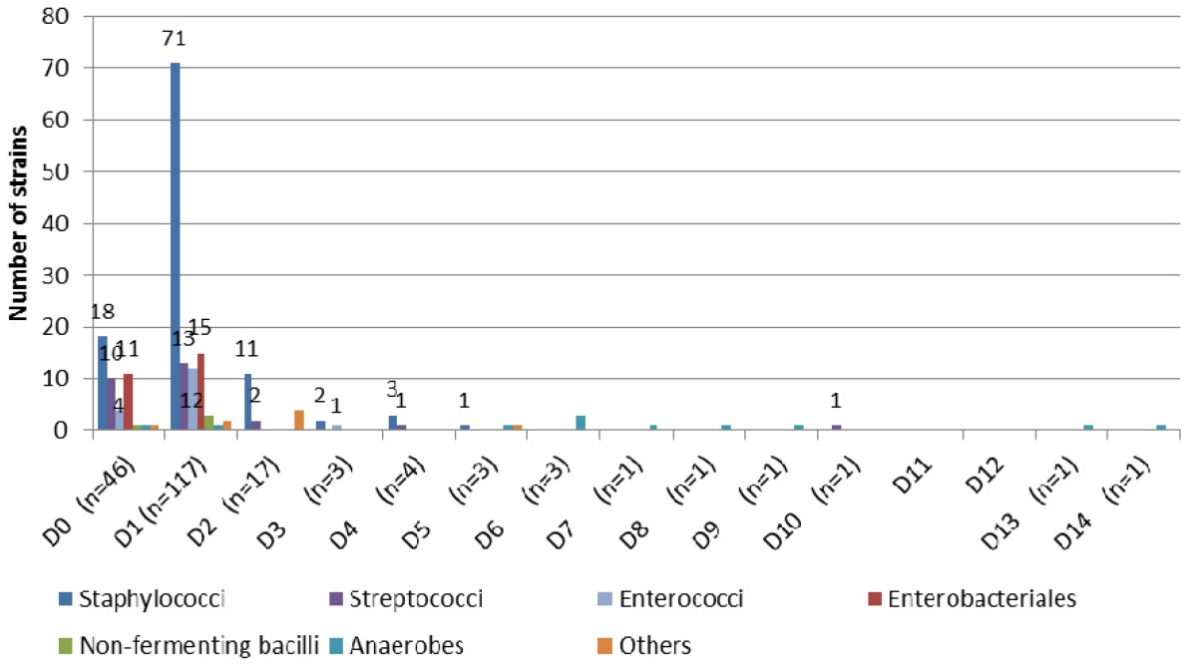
Time of positivity for the 199 strains cultured.

Time of positivity for the 199 cultured strains (from 149 positive surgical procedures) is represented in Figure 1. Using blood culture bottles, 90.5% of the strains (180/199) are detected between D0 and D2. Interestingly, all Gram negative bacilli are detected at D0 or D1. From D3 to D14, 19 strains are cultured, 15 of which are responsible for infections: 4 staphylococci, 2 streptococci, 1 *Enterococcus* sp., 7 *Cutibacterium acnes* and 1 *Cutibacterium avidum*.

The 19 strains cultured from D3 are more precisely described in Table 4 (*C. acnes* and *C. avidum* excluded). Among them, 3 were contaminant strains. The 4 infecting staphylococci and 1 *E. faecalis* had always been cultured in previous surgeries of these patients. Strains of *S. gordonii* and *S. adiacens* were wild-type strains.

## Discussion

Some requirements are commonly admitted for microbiologic studies of PJIs: among them, use of solid culture media and liquid culture media is recommended 2,3,5. However, the optimal duration of incubation of specimens is unknown 2. A duration of 14 days for liquid culture media is commonly used in order to allow isolation of slow-growing organisms such as “micro colony variants” and *C. acnes*, and in cases of suspected PJIs with low virulence organisms 1,4,7.

Until the microbiological results are available, an empirical broad-spectrum antibiotic therapy is usually prescribed in an attempt to cover most Gram-positive cocci and Gram-negative bacilli. Currently in our hospital, we wait until 5 days of culture before modifying this PEAT in accordance with microbiological findings. However, this treatment endangers the patient to select bacterial resistance but also increases both the antibiotic-related adverse effects and the overall cost of the treatment. Thus, accelerate the microbiological diagnosis could help in reducing the duration of broad-spectrum antibiotic therapy and could allow in most cases an oral therapy. Indeed, as soon as the results are available the coverage of Gram-negative rods can be stopped, and the empirical PEAT be focused on Gram-positive cocci bacilli (including methicillin-resistant strains) which represents a significant de-escalade in terms of antibacterial spectrum. If samples do not grow at day 5, the aerobic and anaerobic bottles are incubated until they are positive (maximum day 14). From day 6 to day 14, the most frequently isolated strain is *C. acnes*, which is a bacterium sensitive to antibiotics and does not require a broad-spectrum antibiotic such as daptomycin. Indeed, in our study, only *C. acnes* and *C. avidum* (and one strain of *S. gordonii*) are cultured from D6. In the meantime, semi-automated blood culture systems have been proposed and evaluated for culture of periprosthetic tissue specimens. Several studies pointed out an improvement of sensitivity of detection of organisms causing PJI compared to conventional culture methods 8,9,10,11. Recently, Sanabria *et al*. studied BacT/Alert® Virtuo blood culture bottle system and showed it was as slightly more sensitive than conventional methods 12. Other advantages include a reduced number of specimen required (3 for the most accurate diagnosis in a study performed in 2016) 13, a potential personnel time and labor cost savings 14, and a lower risk of contamination linked to daily inspection of plates. Moreover, it showed higher sensitivity than conventional culture methods in cases with previous antibiotic treatment since bottles contain antimicrobial removal systems 15,16.

Another advantage is a shorter time for microorganism detection compared to conventional culture methods 12,15. In our study, all strains except *C. acnes*, one strain of *C. avidum* and one strain of *S. gordonii* grew within the first five days, accounting for 94.6% of positive surgical procedures. In the study by Peel *et al*., no organism was isolated in aerobic bottles after 7 days of incubation and only *Cutibacterium* sp. grew in anaerobic bottles after 7 days of incubation 8. Minassian *et al*. reported in 2014 that the optimal combination of sensitivity and specificity occurred at day 3 10. In our study, among the 199 strains cultured in 149 surgical procedures, 15 infective strains are cultured from D3, including 7 *C. acnes*, 1 *C. avidum*, 4 staphylococci, 2 streptococci and 1 enterococcus. All Gram negative bacilli are detected with this method at D0 or D1.

These results are of a major importance since it implies that PEAT could reasonably be changed 72h after surgical procedure to a narrower spectrum antibiotic in accordance with microbiological results obtained at D3. For the samples still sterile at D3, an antibiotic therapy effective against *C. acnes* is maintained until D14. Indeed, in our study, we point out that modifying antibiotic therapy in accordance with microbiological results obtained at D3, we take into account all of the Gram-negative bacilli (even at D2). For the infective strains detected from D3, all but one was previously isolated from the patients in other surgeries. The duration of 72h may thus be delayed for patients with previous microbiological history. For the last strain, a *S. gordonii*, it had wild-type antibiotic susceptibility profile and would have been covered by the anti-*Cutibacterium* sp. antibiotherapy proposed. PEAT frequently uses third-generation cephalosporin or cefepime that could be stopped the third day after surgery, allowing a potential oral therapy. In addition to the beneficial for the patient in terms of adverse side effects and lower resistance selection, reduced PEAT duration also represents an economic gain for the hospital.

In our study, *C. acnes* and *C. avidum* were detected by the BacT/Alert® Virtuo blood culture bottle system between the fifth and the fourteenth day of incubation. Therefore, we suggest the need for a prolonged incubation of 14 days of bottles for these anaerobes.

## Conclusion

Our results suggest that the duration of PEAT in patients operated for a PJI may be shorten to three days as Gram-negative rods are unlikely to grow after three days of culture by using BacT/Alert® Virtuo blood culture bottles. This is likely to shorten the overall length of hospital stay, to diminish the occurrence of adverse side effects, and the emergence of antimicrobial resistance. However, coverage of Gram-positive cocci should be maintained for 14 days until the definite culture results are available.

## Authors Contributions

CL and ES conceived and designed the study.

CL and FW obtained original data and analyzed the data.

CD wrote the manuscript.

CL, ES, HM and FW contributed to revise the manuscript.

## Figures and Tables

**Table 1:** Characteristics of 106 patients (216 events) with PJI

Characteristics	
Male, N of patients (%)	53 (50)
Female, N of patients (%)	53 (50)
**Age, years, mean ± SD (range)**	67 ± 12.9 (26-95)
≥75 years old (%)	27 (25.5)
50 - 75 years old	70 (66)
< 50 years old	9 (8.5)
**Location of PJI, N of patients (%)**	
Hip	57
Knee	39
Shoulder	9
Elbow	4
Other prosthesis	4

SD: standard deviation.

**Table 2:** Sterile and positive culture of the 216 series of intraoperative samples

	Bottles : sterile	Bottles : positive
Columbia agar with blood 5% and chocolate agar with polyvitex : sterile	67	58
Columbia agar with blood 5% and chocolate agar with polyvitex : positive	0	91

**Table 3:** Bacterial pathogens isolated from intraoperative samples of PJI in 106 patients

Microorganism	Number of strains (% of the total)	Cultured at day (D)
**Gram-positive cocci**	**151 (75,9)**	**D0-D5**
**Staphylococci**	**107 (53,8)**	
***Staphylococcus aureus***	**44 (22,1)**	
MSSA	41 (20,6)	D0-D1
MRSA	3 (1,5)	D0-D1
**CoNS**	**63 (31,7)**	
*Staphylococcus epidermidis*	38 (19,1)	D0-D4
*Staphylococcus capitis*	10 (5,0)	D1-D3
*Staphylococcus caprae*	4 (2,0)	D1-D2
*Staphylococcus haemolyticus*	2 (1,0)	D2
*Staphylococcus lugdunensis*	6 (3,0)	D1-D5
*Staphylococcus pettenkoferi*	2 (1,0)	D1
*Staphylococcus warneri*	1 (0,5)	D4
**Streptococcus spp.**	**27 (13,6)**	
*Streptococcus adiacens*	2 (1,0)	D2-D4
*Streptococcus anginosus*	1 (0,5)	D1
*Streptococcus agalactiae*	9 (4,5)	D0-D2
*Streptococcus dysgalactiae*	3 (1,5)	D0
*Streptococcus gallolyticus*	2 (1,0)	D0
*Streptococcus gordonii*	2 (1,0)	D1-D10
*Streptococcus mitis/oralis*	3 (1,5)	D0-D1
*Streptococcus pneumoniae*	2 (1,0)	D1
*Streptococcus parasanguinis*	1 (0,5)	D1
*Streptococcus sanguinis*	1 (0,5)	D1
*Streptococcus vestibularis*	1 (0,5)	D1
**Enterococci**	**17 (8,5)**	
*Enterococcus faecalis*	15 (7,5)	D0-D3
*Enterococcus raffinosus*	2 (1,0)	D1
**Gram-negative bacilli**	**29 (14,6)**	**D0-D1**
**Enterobacteriales**	**25 (12,6)**	
*Citrobacter freundii*	1 (0,5)	D0
*Citrobacter koseri*	1 (0,5)	D0
*Enterobacter cloacae*	4 (2,0)	D0-D1
*Escherichia coli*	7 (3,5)	D0-D1
*Klebsiella oxytoca*	1 (0,5)	D0
*Klebsiella pneumoniae*	4 (2,0)	D0-D1
*Morganella morganii*	1 (0,5)	D1
*Proteus mirabilis*	5 (2,5)	D1
*Serratia marcescens*	1 (0,5)	D1
**Non-fermenting bacilli**	**4 (2,0)**	
*Pseudomonas aeruginosa*	3 (1,5)	D0-D1
*Acinetobacter baumannii*	1 (0,5)	D1
**Anaerobes**	**11 (5,5)**	**D0-D14**
*Cutibacterium acnes*	8 (4,0)	D5-D14
*Cutibacterium avidum*	1 (0,5)	D6
*Clostridioides perfringens*	1 (0,5)	D0
*Bacteroides thetaiotaomicron*	1 (0,5)	D1
**Others**	**8 (4,0)**	**D0-D5**
*Bacillus cereus*	2 (1,0)	D0
*Bacillus pumilus*	1 (0,5)	D5
*Corynebacterium striatum*	1 (0,5)	D1
*Micrococcus luteus*	2 (1,0)	D2
Yeast	2 (1,0)	D2
**Total number of strains**	**199 (100)**	

**Table 4:** Strains detected from D3 (*C. acnes* and *C. avidum* excluded)

Patient/Surgery	Strain	Cultured at day	Comments
Patient A /surgery 2	*S. lugdunensis*	5	Known on previous samples
Patient A /surgery 3	*S. lugdunensis*	4	Known on previous samples
Patient B	*E. faecalis*	3	Known on previous samples
Patient C	*B. pumilus*	5	Contaminant strain
Patient D /surgery 2	*S. epidermidis*	4	Known on previous samples
Patient D /surgery 3	*S. epidermidis*	3	Known on previous samples
	*S. gordonii*	10	
Patient E	*S. capitis*	3	Contaminant strain; polymicrobial surgery with *S. aureus*
Patient F	*S. warneri*	4	Contaminant strain; polymicrobial surgery with *S. aureus*
	*S. adiacens*	4	Known on previous samples; polymicrobial surgery with *S. aureus*

## Abbreviations

D: day
DAIR: debridement antibiotic and implant retention
PEAT: post-operative empirical antibiotic therapy
PJI: periprosthetic joint infection
